# Nest Predators and Reproductive Success in the Chinese Francolin (*Francolinus pintadeanus*) Across Two Nature Reserves of Tropical Hainan Island, China

**DOI:** 10.3390/ani15172489

**Published:** 2025-08-25

**Authors:** Qingling Zeng, Yuhan Zhang, Yishuo Ding, He Yang, Yuxin Xu, Guanmian Wu, Xiaodong Rao

**Affiliations:** 1 School of Tropical Agriculture and Forestry, Hainan University, Danzhou 571737, China; 18898971513@163.com (Q.Z.); zyuhan27@163.com (Y.Z.); 18366313798@163.com (Y.D.); xiaoyang123h@163.com (H.Y.); xuyuxin20181029@163.com (Y.X.); 18289697587@163.com (G.W.); 2 Dongfang Natural Resources and Planning Bureau, Dongfang 572699, China; 3 Intelligent Forestry Key Laboratory of Haikou City, Hainan University, Haikou 570228, China

**Keywords:** reproductive success, nest predation, Chinese francolin, infrared camera, artificial nest

## Abstract

Understanding the reproductive ecology of birds and the factors influencing nest predation is essential for developing scientifically sound and effective bird conservation strategies. Certain pheasant species sensitive to environmental changes are vulnerable to threats and face survival pressures such as habitat destruction and human activities. However, research related to their reproductive ecology is lacking. Here for the first time we reported information on breeding biology of the Chinese francolin (*Francolinus pintadeanus*). This study was conducted during the breeding seasons of the Chinese francolin in 2021, 2023, 2024, and 2025, combining traditional survey and infrared camera technology to monitor its reproductive ecology and nest predators in the Datian and Bangxi Reserves and to identify its potential predators through artificial nest experiments. All nests were open-ground nests located at the roots of dwarf shrubs and grasses. Our findings revealed that the breeding season of the Chinese francolin was mainly in March–September, peaking in May; its clutch size was 4.09 ± 1.27 (*N* = 22), reproductive success was 27.27%, and 16 nests were failed; and all failed nests were predated, with abandoned nests accounting for 93.75% of the failed nests. In artificial nest experiments, the predation rates of Datian Reserve and Bangxi Reserve were 70.91% (*N* = 55) and 60.00% (*N* = 30), respectively, with no significant difference in predation rates between the fully covered and exposed groups (Datian: *χ*^2^ = 0.258, *p* = 0.612; Bangxi: *p* = 0.710). Natural nest monitoring and artificial nest experiments on the Chinese francolin identified snakes and the small Indian civet (*Viverricula indica*) as the main predators in Datian Reserve, as well as the greater coucal (*Centropus sinensis*) and wild boar (*Sus scrofa*) as potential predators. In contrast, the main predators in Bangxi Reserve were snakes and rodents. These findings indicate differences in nest predator taxa between the two reserves. We recommend prioritizing the restoration of dwarf scrub vegetation and optimizing the habitat management strategy in these reserves to better protect the breeding habitats of pheasants while promoting long-term stability and continuation of their populations.

## Simple Summary

This study is the first to report on the reproductive biology of the Chinese francolin in the wild. Natural nests of Chinese francolin in two protected areas in Hainan Province were monitored through traditional surveys and infrared camera technology, while artificial nest experiments were conducted to identify major and potential nest predators. The results showed that Chinese francolin breed mainly from March to September each year. All nests were open-ground nests located at the roots of dwarf shrubs and grasses. Breeding success in natural nests was 27.27%, with high rates of nest predation and nest abandonment. Artificial nest experiments showed no significant differences in predation rates between fully covered and exposed groups in the two reserves, and different predator taxa in the two reserves. With the results of this study, we recommend that restoration of dwarf shrub vegetation and optimization of habitat management strategies can protect the breeding habitats of pheasants while promoting the long-term stability and continuation of their populations.

## 1. Introduction

Reproduction is the most crucial component of the avian life history and a key stage determining the dynamics and fate of avian populations [1,2]. Avian reproductive ecology is a critical part of understanding the adaptive mechanisms of species survival, primarily involving research on reproductive behaviors, nest site selection, nest predation pressure, and other aspects [3,4,5]. These studies have elucidated the intrinsic links between avian reproductive strategies and environmental adaptation while also providing a scientific basis for the assessment of avian population dynamics and the formulation of conservation strategies [6,7,8]. As a primary factor contributing to reproductive failure in birds, nest predation has been identified as a crucial selective pressure driving the evolution of avian life-history strategies [9,10,11,12]. Studies have shown that ground-nesting birds often face a higher risk of nest predation compared to other nesting methods [13,14].

Birds of the family Phasianidae are sensitive to environmental changes, and can serve as indicator taxa reflecting the health of the local ecological environment to some extent [15,16,17]. Compared to other birds, pheasants have a higher risk of endangerment [18]. Studies have shown that most Phasianidae species are ground-nesting [19,20], making them more vulnerable to disturbances. Recent reports have found that the pheasant survival rate in Hainan is becoming increasingly threatened due to human hunting activities and habitat destruction [21]. Basic reproduction data and nest predation information serve as important evidence for the formulation of species conservation strategies. However, the skittish behavior of pheasants and their cryptic nesting behavior have made it difficult to study their reproductive ecology, resulting in a relative dearth of relevant research. In fieldwork, camera traps offer advantages such as continuous monitoring and reduced disturbance to wildlife [22,23,24]. This technology has also recently been applied to the reproduction-related research of pheasants, thus providing a new approach and data support for exploring pheasant reproductive ecology [25,26]. For example, Rao et al. (2017) employed infrared cameras to capture the special reproductive behaviors of the Hainan partridge (*Arborophila ardens*) [27]. Artificial nest experiments can minimize the human disturbance to natural nests and compensate for the inadequate sample sizes of field experiments [28,29,30,31]. For example, Rao et al. (2023) combined the use of infrared cameras and artificial nest experiments to explore the reproductive ecology and potential predators of the red junglefowl (*Gallus gallus jabouillei*) [32].

The Chinese francolin (*Francolinus pintadeanus*) is a pheasant of the order Galliformes, family Phasianidae, and genus *Francolinus*, mainly distributed across the Indochinese Peninsula and the southeastern coastal regions of China [33]. Within China, this species is mainly distributed in areas such as Yunnan, Guizhou, Guangxi, and Hainan [34]. The Chinese francolin is classified as a species of Least Concern in its conservation status [35], and is frequently overlooked in species conservation strategies. Hence, there is an even greater paucity of research concerning this species compared to other pheasants. At present, reproduction-related research on the Chinese francolin is limited to the reproductive behaviors and some reproductive biology data recorded by Zhao (2001) and a report by Teng (2022) on the nesting habitats of a few individual Chinese francolins distributed in the Datian National Nature Reserve in Hainan [36,37].

Although tropical regions have the richest avian diversity [38,39,40], current research on the reproductive ecology of tropical birds is scarce [41,42]. As the largest tropical island in China, Hainan Island boasts a unique geographically isolated environment and rich biodiversity [43,44], and is hence an important region for studying the ecology of tropical birds. The Chinese francolin is one of the pheasant species found in this region, and understanding its reproductive information is of great significance to clarifying the ecological characteristics of tropical birds and expanding our knowledge of their life-history strategies. In this study, infrared cameras and artificial nest experiments were employed to survey and monitor the reproductive information and nest predators of the Chinese francolin distributed in the Datian National Nature Reserve (hereinafter referred to as Datian Reserve) and the Hainan Bangxi Provincial Nature Reserve (hereinafter referred to as Bangxi Reserve). It is hoped that our findings will serve to enrich the research on avian life history in tropical regions, as well as to provide scientific evidence for formulating conservation and management measures for this species and promoting habitat restoration for grounding-nesting pheasants.

## 2. Study Areas and Methods

### 2.1. Study Areas Profile

**Figure 1:**
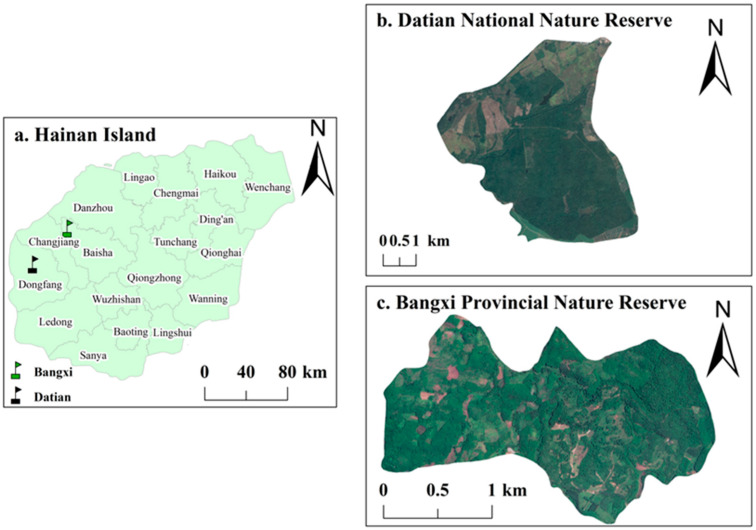
Satellite map of the two study areas.

The study areas consisted of Datian Reserve and Bangxi Reserve (Figure 1). Datian Reserve (19°05′–17′ N, 108°47′–49′ E) is located in Dongfang City in the southwest of Hainan (Figure 1b) at an elevation of 30–80 m, with the reserve covering an area of 1310 ha [32]. Bangxi Reserve (19°22′–24′ N, 109°05′–06′ E) is located in Bangxi Town in the northwest of Baisha County (Figure 1c) at an elevation of 17–80 m, with the reserve covering an area of 361.8 ha [32]. Datian Reserve and Bangxi Reserve are both wildlife nature reserves, whose main purpose is to protect the Hainan Eld’s deer (*Rucervus eldii*) and its habitat. In addition to stable populations of the Chinese francolin, the two reserves also contain its close relative, the red junglefowl [32].

### 2.2. Monitoring of Natural Nests

Field data were collected in Datian Reserve and Bangxi Reserve in March–September of 2021, 2023, 2024, and 2025. Monitoring of the Chinese francolin was carried out at 06:00–09:00 and 16:00–19:00 daily. We searched for the natural nests of the Chinese francolin based on its behaviors and calls in conjunction with the patrols of forest rangers. Any natural nest discovered was GPS-tagged, and the status of the female Chinese francolin (brooding or taking flight) and the nest (nest building, brooding, abandoned) was recorded. To minimize human disturbance, the nests were observed using a telescope once every 7 d. If at least one egg in the nest hatched successfully, successful reproduction was assumed to have occurred; if none of the eggs hatched or were all destroyed, reproductive failure was recorded [45].

**Figure 2:**
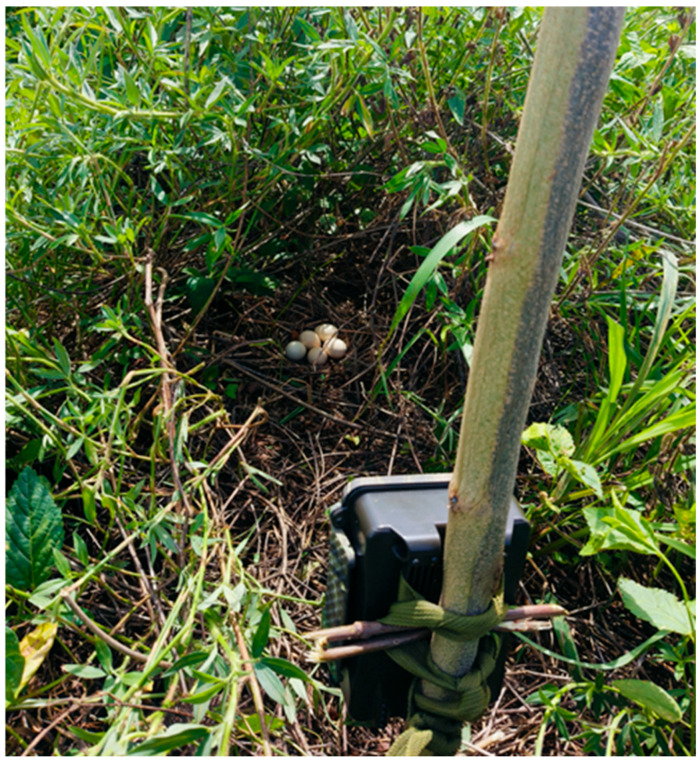
Infrared camera mounting diagram.

An infrared camera was installed for each breeding nest during the incubation period to monitor the fate of the nest and the presence of nest predators. The infrared camera was installed by fixing it to a tree trunk 1–1.5 m away from the nest (Figure 2). In the absence of suitable tree trunks, the infrared camera was mounted on a piling that was suitably camouflaged. The infrared camera was set to record continuously throughout the day, and the shooting mode was set to “photograph + video”; i.e., the camera captured three photographs and 30 s of video each time it was triggered, and the trigger sensitivity was set to “medium” [46]. Following nest predation, the recording was reviewed to document predator type and predation time. For predation events where the predators were undocumented due to instrument failure or other reasons, the type of predator was inferred based on the conditions within and around the predated nest.

After the end of breeding in the natural nests, the following nest parameters were measured: (1) nest long and short diameters (precision: 0.1 cm); (2) nest depth (precision: 0.1 cm); (3) nest materials; (4) distance of nest from water source (precision: 1 m); and (5) shortest distance of nest from roads (precision: 1 m). For natural nests in which reproduction had failed, the following egg parameters were also measured in addition to the parameters above: (1) clutch size; (2) egg long and short diameters; and (3) egg weight. Digital Vernier calipers were used to measure the long and short diameters of Chinese francolin eggs (precision: 0.01 mm), and an electronic scale was used to weigh the eggs (precision: 0.01 g). Nest and nest site parameters were measured using a 5 m measuring tape, and distances exceeding the range of the tape measure were measured using the walking estimation method.

### 2.3. Artificial Nest Experiments

**Figure 3:**
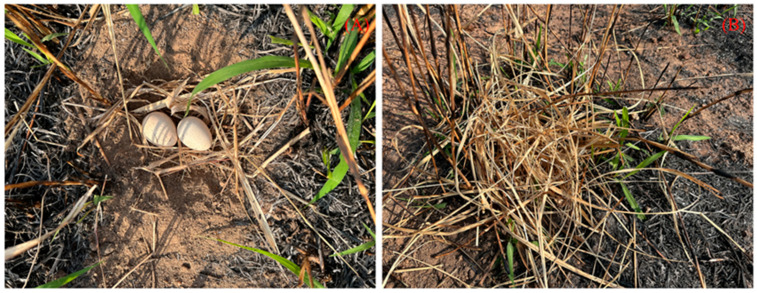
Pictures of artificial nests: ((**A**) eggs are not covered; (**B**) eggs are covered with leaves).

**Figure 4:**
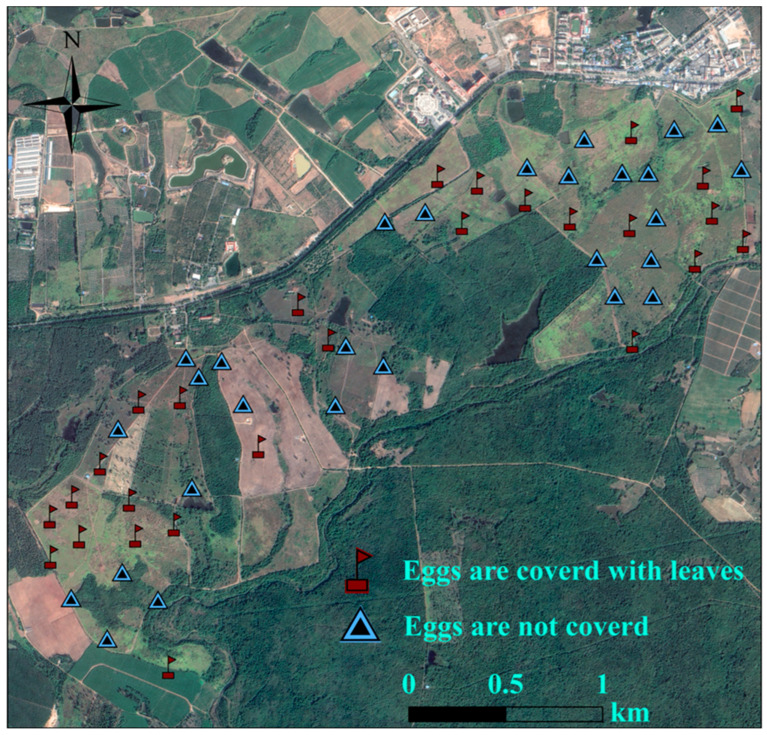
Distribution of artificial nests in Datian National Nature Reserve, Hainan, in 2023.

**Figure 5:**
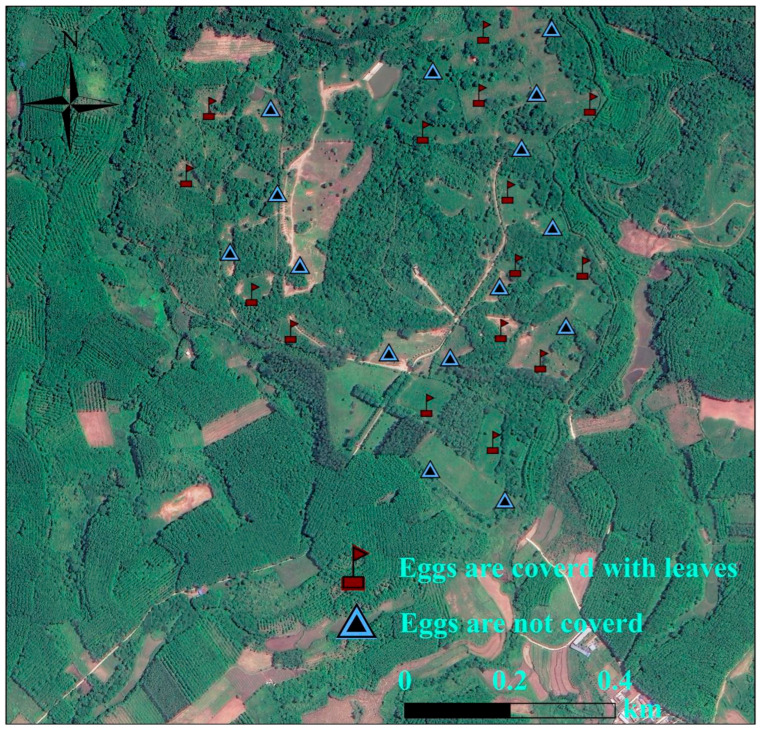
Distribution of artificial nests in Bangxi Provincial Nature Reserve, Hainan, in 2023.

Artificial nest experiments were carried out in Datian Reserve (March–July 2023) and Bangxi Reserve (April–July 2023). Using leaves, twigs, and feathers as nesting materials, artificial nests were constructed by imitating the natural nests of the Chinese francolin, and placed within potential nesting sites [32]. Two unfertilized eggs of domesticated Chinese francolins that were similar in size and color to those of wild Chinese francolins were placed in each artificial nest (Figure 3). Based on whether the experimental eggs were covered with dry leaves, two groups of experiments were set up, namely, the “fully covered” and “non-covered” groups (Figure 3). A total of 55 artificial nests were placed in Datian Reserve, among which 27 were fully covered and 28 were non-covered (Figure 4). A total of 30 artificial nests were placed in Bangxi Reserve, among which 15 were fully covered and 15 were non-covered (Figure 5). In addition, infrared cameras were installed for some of the artificial nests to record the status of nest predation. The experimental period was 21 days, which was based on the natural incubation period of the species, during which a total of 2 examinations were carried out, on days 10 and 21. Documentation of artificial nest status at each inspection. Artificial nests were recorded as predated if at least one egg was damaged or missing, and signs of predation were recorded for each predated nest (no predation traces, with eggshells or predation traces).

### 2.4. Data Analysis

Animals photographed using infrared cameras were identified using the *Chinese Wildlife Manual of Mammals* [47] and *A Checklist on the Classification and Distribution of the Birds of China (Fourth Edition)* [34]. An animal photographed by the same camera more than 30 min apart was considered an independent capture and an individual animal, which we used to calculate the number of animals captured using infrared cameras [32,48].

Chi-squared and Fisher’s exact tests were performed to determine the significance of the differences in the natural nest predation rates and artificial nest predation rates between the two study sites, and in the predation rates of artificial nests among the different treatment groups. All tests were two-tailed. *p* < 0.05 indicated the difference was significant; *p* < 0.01 indicated the difference was highly significant. All data were expressed as mean ± standard deviation (SD). Data analysis was performed using the IBM SPSS 26.0 software (IBM Inc., Armonk, NY, USA).

## 3. Results

### 3.1. Natural Nest Monitoring and Nest Predators

**Figure 6:**
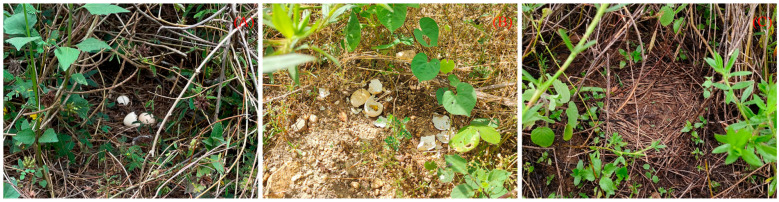
(**A**) Natural nest with successful reproduction; (**B**) predated nest with signs of predation; (**C**) predated nest with no signs of predation.

**Figure 7:**
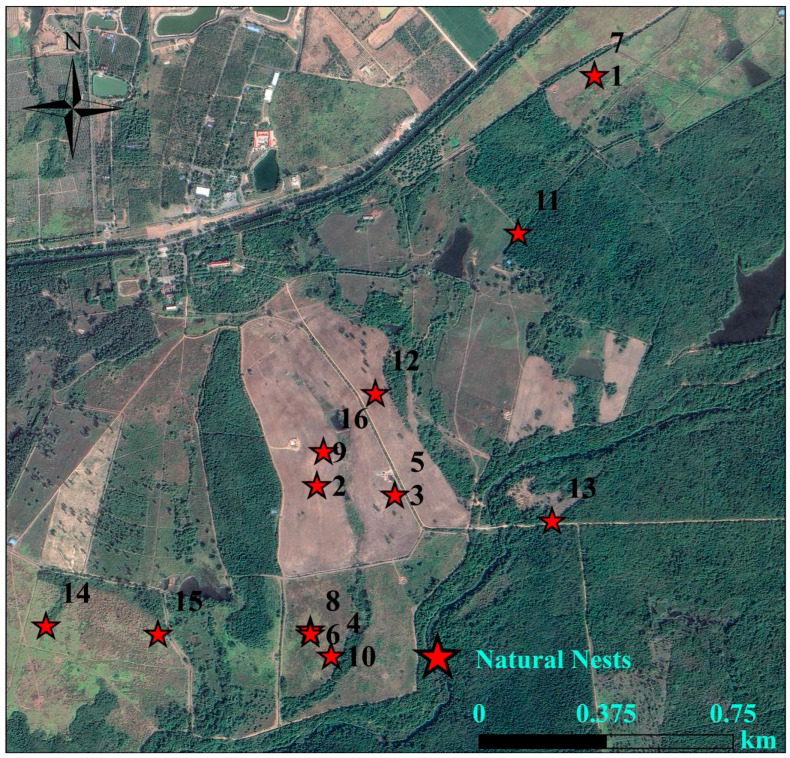
Natural nest sites in Hainan Datian National Nature Reserve in 2021, 2023, 2024, and 2025.

**Figure 8:**
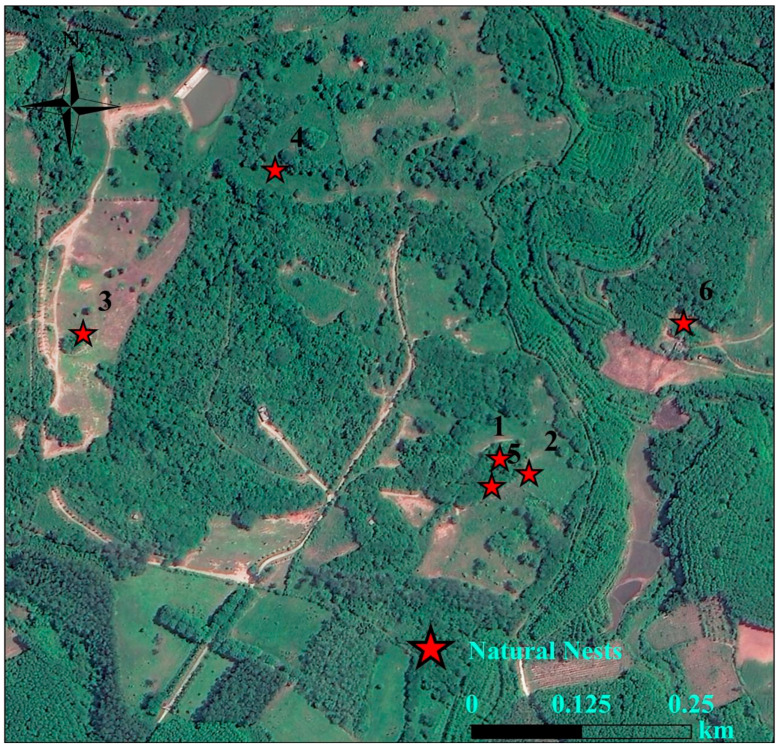
Natural nest sites in Hainan Bangxi Provincial Nature Reserve in 2021, 2023, and 2024.

During the breeding season of 2021, 2023, 2024, and 2025, we discovered a total of 22 Chinese francolin natural nests across the two study sites (Table 1). Among them, 6 nests were reproductively successful (Figure 6), giving a reproductive success rate of 27.27%; and 16 nests were failed, of these, abandoned nests accounted for 93.75% of the failed nests, and all of the failed nests ended up being preyed upon. In addition, 16 nests were found in Datian Reserve (Figure 7) and 6 nests in Bangxi Reserve (Figure 8). The nest predation rate of the Chinese francolin was higher in Datian Reserve compared to Banxi Reserve, but the difference was not significant (81.25% vs. 50.00%, Fisher’s exact test, *p* = 0.283).

**Figure 9:**
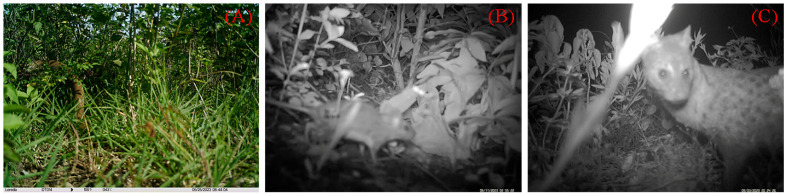
Predators are feeding on eggs in natural nests: ((**A**) oriental rat Snake; (**B**) rodent; (**C**) small Indian civet).

Infrared cameras were installed to monitor 16 natural nests. The video data showed that five eggs in nest 017 were preyed on by an oriental rat snake (*Ptyas mucosus*) (Figure 9A); five eggs in nest 016 were preyed on by a snake (unidentified species); five eggs in nest 018 were preyed on by a small rodent (unidentified species) (Figure 9B); and five eggs each in nests 06 and 08 were preyed on by small Indian civets (*Viverricula indica*) (Figure 9C).

**Figure 10:**
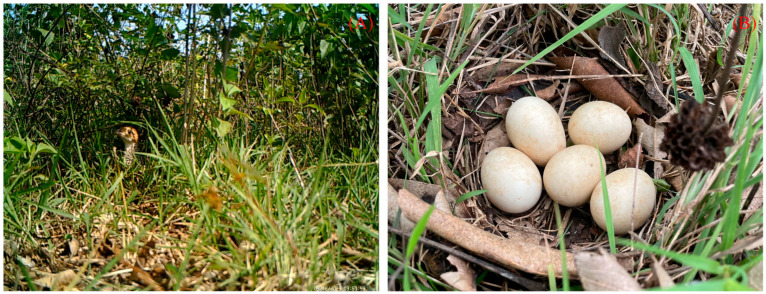
Pictures of natural nests of Chinese francolin: ((**A**) Females brooding; (**B**) Eggs of Chinese francolin).

All natural nests found in this study were open-ground nests without dry leaves or branches covering the eggs (Figure 10B). The discovered nests were all located at the roots of dwarf shrubs and grasses. The nests were oval, shallow pits lined with soft shrubs and grasses, dead twigs, leaves, etc. (Figure 10). The number of natural nests was highest in May (8 nests, accounting for 36.36% of the total number), followed by August (6 nests, 27.27%), June (5 nests, 22.73%), and July (3 nests, 13.64%). The parameters of the eggs and nests in natural nests are shown in Table 2.

### 3.2. Artificial Nest Experiments and Potential Nest Predators

In 2023, of the 55 artificial nests placed in Datian Reserve, 39 were preyed on. Meanwhile, in Bangxi Reserve, 18 of the 30 artificial nests placed were preyed on. The predation rate in Datian Reserve was higher than that in Bangxi Reserve, but the difference was not significant (70.91% vs. 60.00%, Pearson’s Chi-squared Test, *χ*^2^ = 1.046, *p* = 0.306).

Among the 28 non-covered artificial nests placed in Datian Reserve, 19 were preyed on; among the 27 fully covered nests, 20 were preyed on. In Datian Reserve, the predation rates of non-covered and fully covered nests did not differ significantly (67.86% vs. 74.07%, Pearson’s Chi-squared Test, *χ*^2^ = 0.258, *p* = 0.612). Among the 15 non-covered artificial nests placed in Bangxi Reserve, 8 were preyed on; among the 15 fully covered nests, 10 were preyed on. In Bangxi Reserve, the predation rates of non-covered and fully covered nests did not differ significantly (53.33% vs. 66.67%, Fisher’s exact test, *p* = 0.710).

**Figure 11:**
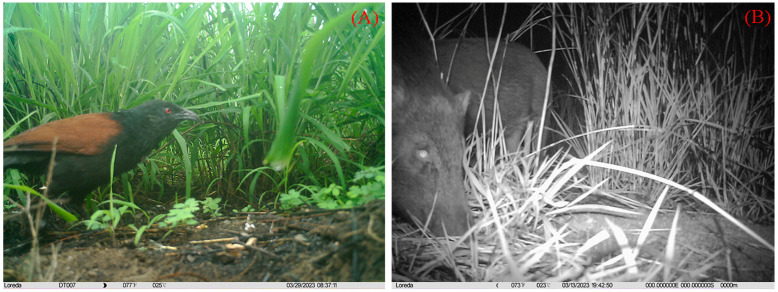
Predators are feeding on eggs in artificial nests: ((**A**) greater coucal; (**B**) wild boars).

**Figure 12:**
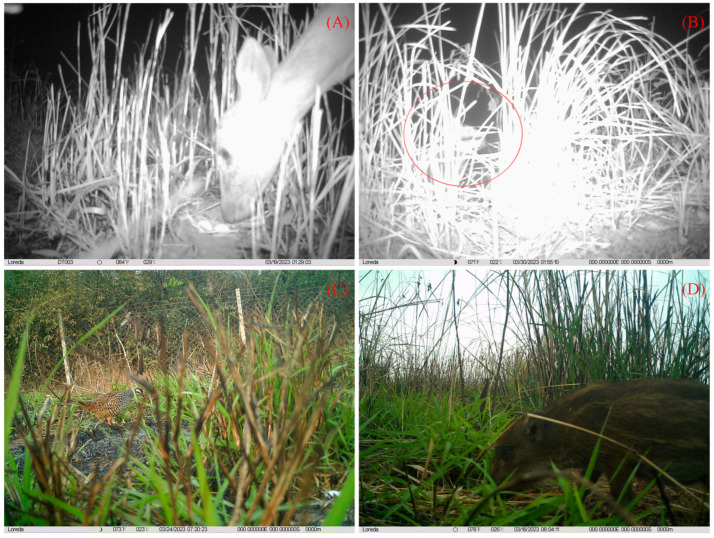
Visitors near artificial nests: (**A**) Hainan Eld’s deer; (**B**) Hainan Hare (the red circle indicates); (**C**) Chinese francolin; (**D**) wild boar cub.

In the artificial egg experiments, three predation events were captured using infrared cameras, including one avian predator (greater coucal, *Centropus sinensis*) (Figure 11A) and two mammalian predators (small Indian civet; and wild boar, *Sus scrofa*) (Figure 11B), all of which were captured in Datian Reserve. In addition, other visiting animals were photographed, including the Hainan Eld’s deer, Hainan hare (*Lepus hainanus)*, and Chinese francolin in Datian Reserve, and the Hainan Eld’s deer and a few rodents in Bangxi Reserve (Figure 12).

## 4. Discussion

### 4.1. The Overview of Chinese Francolin’s Breeding

During the breeding season in 2021, 2023, 2024, and 2025, a total of 22 natural nests of the Chinese francolin were discovered, with a reproductive success rate of 27.27%, all failed nests were predated and abandoned nests accounted for 93.75% of failed nests. We found that Chinese francolin parents showed a very high rate of nest abandonment when disturbed, and eggs were highly likely to be preyed upon in abandoned nests.

### 4.2. The Nest Predation and Nest Predators of Chinese Francolin

This study found that nest predation is a key factor contributing to nest failure. By monitoring natural and artificial nests using infrared cameras, we were able to document a wide range of nest predators and visitors. Six predators were photographed in Datian Reserve, including three mammals, two birds, and one reptile. The visitors in Datian Reserve were mainly mammals, while only Hainan Eld’s deer and rodents were documented in Bangxi Reserve. Infrared camera data suggest that mammals were the main predators of natural and artificial nests. However, the majority of predation events were unrecorded. Different predators leave different traces after preying on nests. For example, rodents will leave behind eggshell fragments after predation, whereas snakes often leave no traces behind [49,50]. In Datian Reserve, no traces were found in the majority of predated nests; in Bangxi Reserve, traces of rodent gnawing were found in some artificial nest, while others had no traces. Therefore, we speculate that in addition to the mammals documented, the major nest predators in Datian Reserve also included snakes, whereas the predators in Bangxi Reserve were predominantly rodents and snakes (based on our team’s unpublished data). However, further precise field surveys and monitoring are needed in the future to confirm more specific predator information. The predator taxa of the Chinese francolin were similar to those of the sympatric red junglefowl [32]. However, the predator taxa of the Chinese francolin also included small-to-medium-sized and medium-to-large-sized mammalian predators. This difference may be closely associated with the nest site selection strategies of the two species. Large-sized predators are generally more vigilant toward humans and tend to avoid areas with frequent human activity [51,52,53,54]. Therefore, in contrast to the red junglefowl, which prefers to build its nest closer to roads in the vicinity of human activities, the Chinese francolin, which chooses to build its nest further from roads, may face a greater predation risk posed by mammals.

This study found that for both natural and artificial nests, the nest predation rate in Datian Reserve was slightly higher compared to Bangxi Reserve. Other studies have reported that nest predators and nest predation rates may vary, even between highly similar areas [55,56]. For instance, Maag et al. (2022) compared the nest predation rates of the ground-nesting wood warbler (*Phylloscopus sibilatrix*) across multiple breeding areas and found that the nest predation rate was positively correlated with the forest area of the breeding sites [57]. The predation amount of sables on the nests of grouse (*Tetrao urogallus*) decreased with the increase of the density of agricultural land, and increased from the forest to the forest [58]. Hence, the disparities in predator types under different spatial scales may be one of the major factors contributing to different nest predation rates. Datian Reserve covers a larger area compared to Bangxi Reserve, and there were differences in the type and threat level of nest predators across the two study sites. The predators in Bangxi Reserve were predominantly small-sized predators, such as the oriental rat snake and rodents, whereas Datian Reserve had a richer variety of predators, encompassing not only small-sized predators, but also medium-to-large-sized predators, such as the small Indian civet and wild boar. Furthermore, Bangxi Reserve is predominantly surrounded by rubber plantations, with fewer dwarf shrubs, whereas Datian Reserve is mainly surrounded by residential areas and farmland. This difference in edge complexity between the two study sites may also be one of the causes for the discrepancies in the type and number of predators.

### 4.3. The Breeding Strategy and Anti-Prey Strategy of Chinese Francolin

Our results from monitoring the natural nests of the Chinese francolin in Datian Reserve revealed that its reproductive success rate was 18.75%, which was far lower than the results by Teng (2022) in the same region (75.00%), whereas the nest abandonment rate (81.25%) was slightly higher than that reported by Teng (2022) (66.67%) [37]. The sample size for Chinese francolin nests was relatively small in the study by Teng (2022), and hence our study results have greater validity [37]. Previous studies have found that pheasants have very high vigilance, and are even more sensitive during the breeding season [17,59,60,61]. Our study also found that Chinese francolins were highly vigilant during the breeding season, with most parents opting to abandon their nests when disturbed. Some tropical birds adopt the double or multiple brooding strategy to improve their reproductive success rate [62,63,64,65]. Our study showed that the Chinese francolin exhibited very high nest abandonment and nest predation rates. However, breeding nests of the Chinese francolin were also discovered in August–September. Hence, we speculate that this species may adopt a multiple brooding strategy, but this requires further investigation and verification. The monitoring results of Chinese francolin natural nests and artificial nest experiments indicated that the nest predation rates were relatively high, thus confirming the high nest predation pressure faced by birds in tropical regions [66], especially among ground-nesting birds [67].

Our findings on the artificial nest experiments indicate that the nest predation rates of both study sites were significantly higher than the results reported by Rao et al. (2023) in the same region [32]. This difference may have been due to the following factors: First, the difference in the study time period may have led to changes in the intensity of predator activities [68,69]. The two experiments were conducted 2–3 years apart, during which the activity patterns or population of predators may have undergone significant changes. Second, the difference in nest site selection may have also contributed to differences in predation rates [70,71,72]. Red junglefowl tend to build nests in areas close to roads, which may have reduced predation rates due to the indirect refuge resulting from human activities [32]. In both studies, the layout of artificial nests strictly adhered to the habits of the target species. Hence, the predation rate of the artificial nests followed a similar trend to that of the natural nests, with both showing higher nest predation rates compared to the previous study.

However, an unexpected finding was whether covering the experimental eggs with leaves had no impact on the predation rate of the artificial nests. The primary reason contributing to this finding may be related to the predator’s mechanism involving the use of multi-sensory cues when searching for prey. Studies have shown that snakes, rodents, felines, and other major predators do not rely solely on visual cues during predation but instead employ a combination of multi-sensory information, including olfaction and audition, to search and locate prey [73,74,75]. Therefore, within the effective search range of predators, coverage (i.e., a visual camouflage) had a limited impact on reducing the predation rate. Furthermore, birds in tropical regions generally face relatively high nest predation pressure [64,66,76]. Hence, in an environment with a high predator density, simple egg concealment measures may be attenuated by environmental factors.

The niche differentiation theory posits that closely related sympatric species must be differentiated in at least one niche dimension to achieve the partitioning of limited resources and reduce interspecific competition, thereby maintaining a long-term stable co-existence [77,78,79,80]. In this study, the breeding season of the Chinese francolin was March–September, peaking in May. In contrast, the breeding season of a closely related sympatric species, the red junglefowl, is in March–July, peaking in April [32]. These two sympatric pheasants may have diverged with respect to their temporal niche. This is consistent with the results of Yu et al. (2011), who analyzed and compared the ecological distribution and spatial niche of pheasants in the karst area of southwest Guangxi, China [81]. They revealed that the red junglefowl occupied the widest spatial niche, whereas the Chinese francolin occupied the narrowest. In addition, our study showed that the nest sites of the Chinese francolin were further from water sources and roads compared to the red junglefowl [32]. The red junglefowl preferred to nest in habitats such as tropical deciduous rainforests, shrub-grasslands, and artificial grasslands [82], whereas our study found that the sympatric Chinese francolin mainly nested in habitats with dwarf shrubs and grasses. These results suggest that the two types of pheasants differed to some extent in terms of their breeding phenology and nest site selection, which further supports the hypothesis that closely related sympatric species achieve co-existence through niche differentiation.

Compared to the Chinese francolin, another pheasant species found in Hainan— the Hainan partridge—exhibited a different nest-building strategy, which involved covering its eggs completely with dry leaves and twigs [27]. The nest predation rate of this species (20.00%) is far lower than that of the open-nesting Chinese francolin, and this finding is inconsistent with our results in the artificial nest experiments. This disparity may have stemmed from the differences in nest structural features between the two species. Our study demonstrated that the nests of the Chinese francolin are characterized by a wide and shallow structure, which is more easily detected by predators through smell. Furthermore, when predators are foraging, their body movements will disturb the surrounding cover of leaves, and the shallow placement of eggs means that they are easily exposed, even in fully covered artificial nests. In contrast, the nests of the Hainan partridge had a narrower and deeper arch feature, making their eggs less easily detectable, which significantly reduced their nest predation risk [27].

## 5. Conclusions

The survival of pheasants in Hainan is threatened by illegal hunting activities and the gradual shrinking of their habitats [21]. Understanding the adaptability of ground-nesting pheasants to habitat fragmentation and the characteristics of their reproductive ecology has significant implications for the formulation of effective conservation measures. Although the Chinese francolin is listed as a species of Least Concern [35], its conservation is frequently overlooked, with far less research compared to other pheasants. Here for the first time we reported information on breeding biology of the Chinese francolin in the wild. This study showed that the Chinese francolin has a preference for nesting at the roots of dwarf shrubs and grasses and was highly sensitive to external disturbances. The two study sites, Datian Reserve and Bangxi Reserve, are both wildlife nature reserves that primarily serve to protect the Hainan Eld’s deer and its habitat. When improving the suitability of habitats for the Hainan Eld’s deer in the two reserves, the primary measure adopted in Datian Reserve was regeneration through controlled burning, while that in Bangxi Reserve was plowing. The improvement measures in both sites have caused varying extents of damage to dwarf shrub vegetation. Furthermore, stable populations of wild boars were found in both sites, which can exert a substantial destructive effect on the ground structure and wildlife habitats [83,84]. These factors have contributed to the destruction of the Chinese francolin’s habitats, which may have led to a decrease in its population density. Therefore, when adopting improvement measures in nature reserves to protect the Hainan Eld’s deer and its habitats, we recommend fully considering the potential impact on the reproduction of ground-nesting pheasants. Specific measures include promoting the restoration of dwarf shrub vegetation in existing environments, enhancing the corresponding regulations for nature reserve management, and minimizing the disturbance of human activities on habitats.

## Figures and Tables

**Table 1:** Active nests of Chinese francolin (*Francolinus pintadeanus*) in Datian and Bangxi Nature Reserves in 2021, 2023, 2024, and 2025.

Nest	Site	Date Found	Clutch Size	Observation and Nest Fate
01 *	DT	31 May 2021	5	No adult, Nest abandoned, Predated
02 **	DT	31 May 2021	5	No adult, Five Nestlings hatched
03 **	DT	23 May 2023	3	No adult, Three Nestlings hatched
04 **	DT	7 May 2023	3	No adult, Three Nestlings hatched
05 *	DT	11 August 2023	4	No adult, Nest abandoned, Predated
06 *	DT	11 August 2023	5	No adult, Nest abandoned, Predated by small Indian civet
07 *	DT	16 August 2023	5	No adult, Nest abandoned, Predated
08 *	DT	16 August 2023	5	No adult, Nest abandoned, Predated by small Indian civet
09 *	DT	30 August 2023	5	No adult, Nest abandoned, Predated
10 *	DT	30 August 2023	5	No adult, Nest abandoned, Predated
11 *	DT	13 June 2024	5	No adult, Nest abandoned, Predated
12 *	DT	26 June 2024	4	No adult, Nest abandoned, Predated
13 *	DT	24 June 2024	5	No adult, Nest abandoned, Predated
14	DT	8 July 2024	1	No adult, Nest abandoned, Predated
15 *	DT	8 July 2024	5	No adult, Nest abandoned, Predated
16 *	DT	10 June 2025	5	Adult flew away, Nest abandoned, Predated by a snake
17 *	BX	14 May 2023	5	No adult, Predated by oriental rat snake
18 *	BX	3 June 2023	5	No adult, Nest abandoned, Predated by rodent
19 **	BX	8 May 2023	3	No adult, Three Nestlings hatched
20 **	BX	13 May 2023	2	No adult, Two Nestlings hatched
21 **	BX	11 May 2024	2	No adult, Two Nestlings hatched
22 *	BX	19 May 2024	3	No adult, Nest abandoned, Predated

DT, Datian National Nature Reserve; BX, Bangxi Nature Reserve. * The nest was monitored by infrared camera; ** The nest had finished breeding when discovered.

**Table 2:** Egg parameters and nest parameters of natural nests of the Chinese francolin.

Parameter Type	Parameter	Value	***N***
Egg parameters	Clutch size	4.09 ± 1.27	22
	Weight (g)	18.67 ± 5.49	10
	Long diameter (mm)	38.32 ± 5.08	10
	Short diameter (mm)	30.26 ± 3.12	10
Nest parameters	Long diameter (cm)	15.60 ± 3.58	13
	Short diameter (cm)	12.09 ± 1.93	13
	Depth (cm)	3.65 ± 0.76	13
	The distance of natural nests from roads (m)	66.51 ± 47.70	22
	The distance of natural nests from water source (m)	212.50 ± 142.85	22

## Data Availability

Further information on the data included in this study is available from the corresponding author upon reasonable request.
